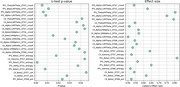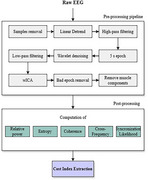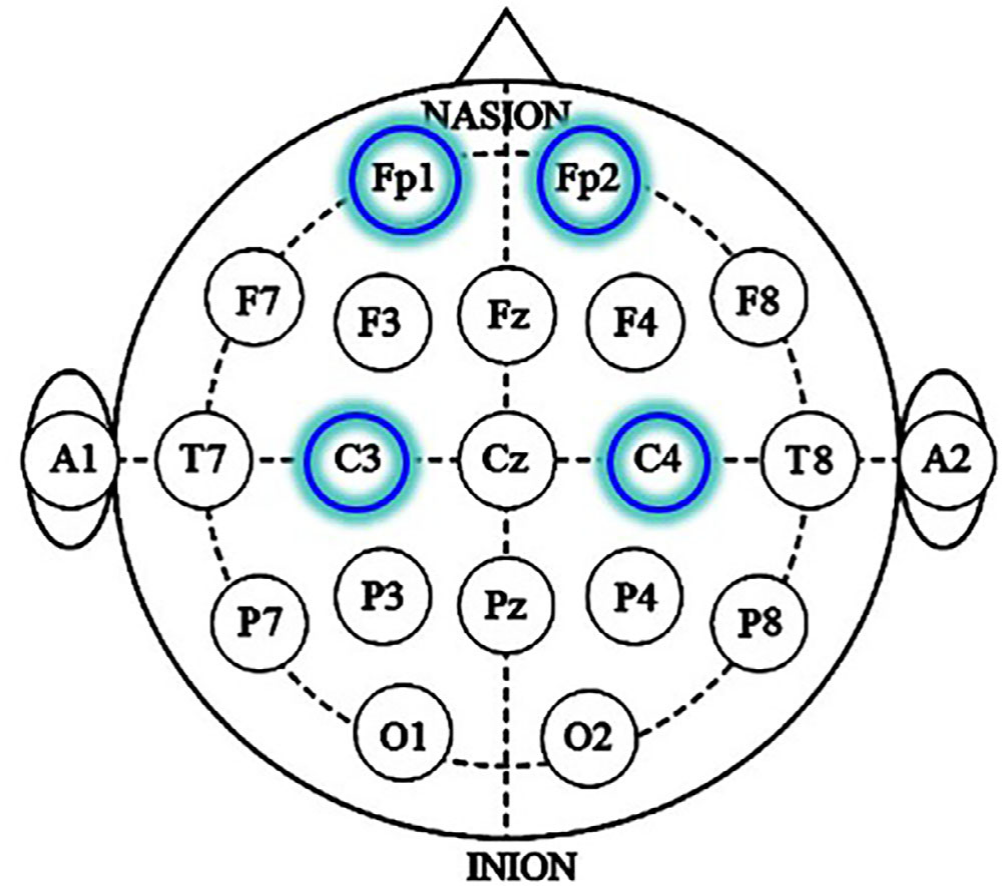# Quantitative EEG obtained from Dual‐Task paradigms for the study of preclinical populations at risk of Alzheimer Disease

**DOI:** 10.1002/alz70856_103932

**Published:** 2025-12-24

**Authors:** Juliana Moreno Rada, Luisa María Zapata Saldarriaga, Elkin Garcia‐Cifuentes, David Fernando Aguillón Niño, María José Hidalgo Ramírez, Alejandro Guerrero, John Fredy Ochoa Gómez

**Affiliations:** ^1^ Universidad de Antioquia, Medellín, Antioquia, Colombia; ^2^ Grupo Neuropsicología y Conducta GRUNECO, Medellín, Antioquia, Colombia; ^3^ Semillero Neurociencias Computacionales NeuroCo, Medellín, Antioquia, Colombia; ^4^ Grupo de Neurociencias de Antioquia GNA, Medellin, Antioquia, Colombia; ^5^ School of Medicine, University of Antioquia, Medellín, Antioquia, Colombia; ^6^ Semillero de Investigación SINAPSIS, Medellín, Antioquia, Colombia; ^7^ Grupo Neuropsicología y Conducta GRUNECO, Universidad de Antioquia, Medellín, Antioquia, Colombia; ^8^ Grupo de Neurociencias de Antioquia, Universidad de Antioquia, Medellin, Antioquia, Colombia; ^9^ Grupo de Neurociencias de Antioquia, Facultad de Medicina, Universidad de Antioquia, Medellín, Colombia; ^10^ School of Engineering, University of Antioquia, Medellín, Antioquia, Colombia

## Abstract

**Background:**

Colombia hosts the largest Autosomal‐Dominant Alzheimer's Disease (ADAD) kindred caused by the PSEN1‐E280A genetic variant, with full penetrance. This ADAD is well‐characterized in terms of clinical and biomarker research, but Electroencephalography (EEG) as a biomarker is still under development. EEG facilitates understanding brain function and electrophysiological changes in ADAD. Dual‐task (DT) paradigms, particularly during gait and upper extremity function, are useful biomarkers to determine cognitive impairments, due to neurodegenerative disorders such as Alzheimer's disease. EEG and DT are non‐invasive, portable, and feasible in outpatient settings, making them highly accessible for clinical practice.

**Method:**

The study sample includes two groups, asymptomatic carriers (*n* = 37) and non‐carriers (*n* = 42) of the PSEN1‐E280A variant. EEG data were recorded during a dual‐task paradigm involving two tasks. The first task was a motor task (single‐task) involving upper extremity flexion‐extension. The second was a motor‐cognitive task (dual‐task), which included subtraction by 7 (DTS7), by 1 (DTS1) or verbal fluency with animals (DTAN). EEG signals were collected using the OpenBCI system from frontal and central regions. Data were processed and spectral and neural dynamics features were extracted. Dual‐task cost (DTC) was calculated as the difference between metric values under dual‐task and single‐task conditions, computed across channels and frequency bands for each feature. The extracted metrics were statistically analyzed using Cohen's‐D effect size and Mann‐Whitney U‐tests to assess differences.

**Result:**

The most notable effect sizes were observed in entropy for Beta1 and Beta3 bands in frontal regions for the DTS7 and DTS1 tasks, Beta2 relative power in C4 during DTAN, Theta modulation in the Theta and alpha‐1 bands in frontal regions during DTS7, and Alpha2 modulations in the same task.

**Conclusion:**

The proposed experiment effectively extracted brain information that differentiates between carriers and non‐carriers of the PSEN1‐E280A variant. DTC values in the alpha band (frontal and central regions) and the beta band (central regions) exhibited larger effect sizes, suggesting that these areas are sensitive to preclinical cognitive and motor differences. The experimental paradigm and data‐processing pipeline ensured the acquisition of EEG data with meaningful neurological information. This combination of novel biomarkers enhances non‐invasive and portable tools for identifying ADAD carriers during preclinical stages.